# Clinical Experience of Personalized Phage Therapy Against Carbapenem-Resistant *Acinetobacter baumannii* Lung Infection in a Patient With Chronic Obstructive Pulmonary Disease

**DOI:** 10.3389/fcimb.2021.631585

**Published:** 2021-02-26

**Authors:** Xin Tan, Huaisheng Chen, Min Zhang, Ying Zhao, Yichun Jiang, Xueyan Liu, Wei Huang, Yingfei Ma

**Affiliations:** ^1^ Shenzhen Key Laboratory of Synthetic Genomics, Guangdong Provincial Key Laboratory of Synthetic Genomics, CAS Key Laboratory of Quantitative Engineering Biology, Shenzhen Institute of Synthetic Biology, Shenzhen Institute of Advanced Technology, Chinese Academy of Sciences, Shenzhen, China; ^2^ Department of Critical Care Medicine, Shenzhen People’s Hospital (The Second Clinical Medical College of Jinan University, The First Affiliated Hospital of South University of Science and Technology), Shenzhen, China; ^3^ Shenzhen People’s Hospital, Shenzhen Institute of Respiratory Diseases, Shenzhen, China; ^4^ Bacteriology and Antibacterial Resistance Surveillance Laboratory, Shenzhen People’s Hospital (The Second Clinical Medical College, Jinan University, The First Affiliated Hospital, Southern University of Science and Technology), Shenzhen, China; ^5^ Department of Geriatrics, Shenzhen People’s Hospital (The Second Clinical Medical College of Jinan University, The First Affiliated Hospital of South University of Science and Technology), Shenzhen, China

**Keywords:** carbapenem-resistant *Acinetobacter baumannii*, lung infection, personalized phage therapy, phage, endotoxin

## Abstract

Overuse of antibiotics in clinical medicine has contributed to the global spread of multidrug-resistant bacterial pathogens, including *Acinetobacter baumannii*. We present a case of an 88-year-old Chinese man who developed hospital-acquired pneumonia caused by carbapenem-resistant *A. baumannii* (CRAB). A personalized lytic pathogen-specific single-phage preparation was nebulized to the patient continuously for 16 days in combination with tigecycline and polymyxin E. The treatment was well tolerated and resulted in clearance of the pathogen and clinical improvement of the patient’s lung function.

## Introduction

Infections caused by carbapenem-resistant *Acinetobacter baumannii* (CRAB) impose a major challenge in clinics (Perez et al., 2007; Maragakis and Perl, 2008; Wong et al., 2017). The most common CRAB infections, i.e., hospital-acquired pneumonia (HAP) and bloodstream infections, are often associated with extremely high mortality (Isler et al., 2019). The World Health Organization (WHO) designated CRAB as the critical-priority pathogen in the list of “priority pathogens”, a group of bacteria that poses the greatest threat to human health. Novel strategies to treat CRAB infections are urgently needed (Tacconelli et al., 2018).

There have been increased initiatives to develop bacteriophage (phage) as an alternative or supplement to antibiotics in treating CRAB infections (Gordillo Altamirano and Barr, 2019; Kortright et al., 2019). Phage therapy has been applied in personalized therapy, and several examples of the successful treatment of infections caused by CRAB have been reported (Schooley et al., 2017; LaVergne et al., 2018). However, to the best of our knowledge, no case of lung infection with CRAB treated with phage therapy has been reported yet. Here, we present our clinical experience regarding phage therapy to treat CRAB lung infection in an 88-year-old Chinese man.

## Materials and Methods

### Bacterial Strains

Clinical isolates were obtained from routine microbiological cultures of clinical samples: blood and bronchoalveolar lavage fluid (BALF). *A. baumannii* isolates were maintained in Luria-Bertani (LB) broth (Huankai Microbiol, Guangzhou, China) and stored in 15% glycerol at -80°C. Surveillance BALF cultures were screened for carbapenemase-producing (CP) *Enterobacteriales* by enrichment of non-selective LB medium and subsequent inoculation in selective chromogenic mSuperCARBA plate (CHROMagar, Paris, France). Creamy colonies were considered as the CP *A. baumannii* and re-identified by matrix-assisted laser desorption/ionization mass spectrometry (bioMérieux, Marcy-l’Étoile, France). The antimicrobial susceptibility testing was performed with the VITEK-2 compact system (bioMérieux, Marcy-l’Étoile, France). The results were interpreted based on the guidelines published by the Clinical and Laboratory Standards Institute (Institute CaLS, 2016).

### Isolation of Phages

Phages used for this treatment were isolated and prepared at Shenzhen Institutes of Advanced Technology. Phages were isolated from various environmental samples by using routine isolation techniques, as previously described (Chen et al., 2018), except the cultures were incubated at 37°C. Briefly, *A. baumannii* clinical isolates obtained from the patient’s BALF culture were used to isolate and propagate pathogen-specific phages. Following isolation, the phages were triple plaque-purified on their respective host bacterium. Finally, small-scale phage amplification on their corresponding host bacterium was performed to prepare the *A. baumannii*-specific phage library, which was subsequently stored at 4°C until required.

### Phage DNA Extration, Genome Sequencing, and Assembly

Phage particles were precipitated with 10% polyethylene glycol 8,000 (PEG 8000) at 4°C overnight, centrifuged at 10,000x g for 15 min, and subsequently suspended in SM buffer (100 mM NaCl, 8 mM MgSO4, 50 mM Tris-HCl), then the concentrated phage particles were treated using DNase I and RNase A (New England BioLab, Massachusetts, USA) to remove bacterial nucleic acids, genomic phage DNA was extracted with Lambda Bacteriophage Genomic DNA Rapid Extraction Kit (Aidlab, Beijing, China) following manufacturer’s protocol (Chen et al., 2020). Whole-genome sequencing was performed at the Tianjing Sequencing Center (Novogene, Beijing, China) using the Illumina HiSeq system (Illumina Inc., San Diego, CA, USA). Reads were assembled with SOAPdenovo2 (Luo et al., 2012). The resulting contigs were uploaded into the RAST server using the RASTtk annotation workflow (Aziz et al., 2008). Putative functions of the ORFs were further identified with Blast-P based on amino acid sequences. A circular map of the phage was depicted using the CGView Server (Grant and Stothard, 2008).

### Transmission Electron Microscopy

20 ul of phage suspension were placed onto a copper grid with carbon film, left for 3 to 5 min, and the excess liquid was removed using filter paper. Staining was performed with 2% phosphotungstic acid for 1 to 2 min, and the cuprum grids were observed under Transmission Electron Microscopy (HT7800, Hitachi, Tokyo, Japan).

### Propagation and Purification of Therapeutic Phages

The phage preparations were processed in two different ways. The first batch phage preparation was purified with Amicon Ultra-15 centrifugal filter units (MWCO 100 K Da, Merck Milipore, Massachusetts, USA) (Bonilla et al., 2016). The second phage preparation was purified in a cesium chloride density gradient as previously described (Bachrach and Friedmann, 1971) and then dialyzed using a Spectra/Por RC membrane (MWCO 20 K Da, Spectrum, **New Jersey,** USA) in phosphate-buffered saline to remove cesium chloride. Dialysis reduced the cesium concentration to less than 210 parts per billion, as detected by inductively coupled plasma mass spectrometry (ICP-MS) (Dedrick et al., 2019). Phages were then sterilized through 0.22-µm filters. Then phages were titrated and evaluated for endotoxin with an End-point Chromogenic Endotoxin Test Kit (Bioendo, Xiamen, China). Phage preparations were subsequently stored at 4°C until required. The resulting titers and endotoxin levels were 5x10^8^ PFU/ml and 10^5^ EU/ml, respectively, for the first batch, and 5x10^10^ PFU/ml and 1.3x10^4^ EU/ml for the second one.

### Phage Therapy

The phage preparation was diluted with saline solution to 5 ml and administrated to the patient by a vibrating mesh nebulizer (Aerogen) every 12 h (about 30 min for each dose), except for the first 2 doses, which were given once daily. The first batch phage preparation was used initially for 13 days, then the second phage preparation was administrated for the rest time of phage therapy. See more details in the Results section.

### Phage Quantification in Patient Samples

BALF samples were collected for measuring phage counts as follows: 0.5 ml sample was transferred to a 1.5 ml Eppendorf tube and 0.5 ml phage buffer was added. After vortexing, the sample was passed through a 0.22 µm filter, serially diluted, and 10 µl added to *A. baumannii* agar overlays.

## Results

### Patient Clinical History

In June 2018, an 86-year-old Chinese man was admitted to our hospital for exacerbation of chronic obstructive pulmonary disease (COPD). The medical history of the patient was documented with a long history of type-2 diabetes. The patient suffered recurrent episodes of lung infections associated with repeated use of mechanical ventilation over the last 2 years during hospitalization. After resuscitation from cardiac arrest in April 2019, the patient had difficulty in weaning from mechanical ventilation.

**Figure 1:**
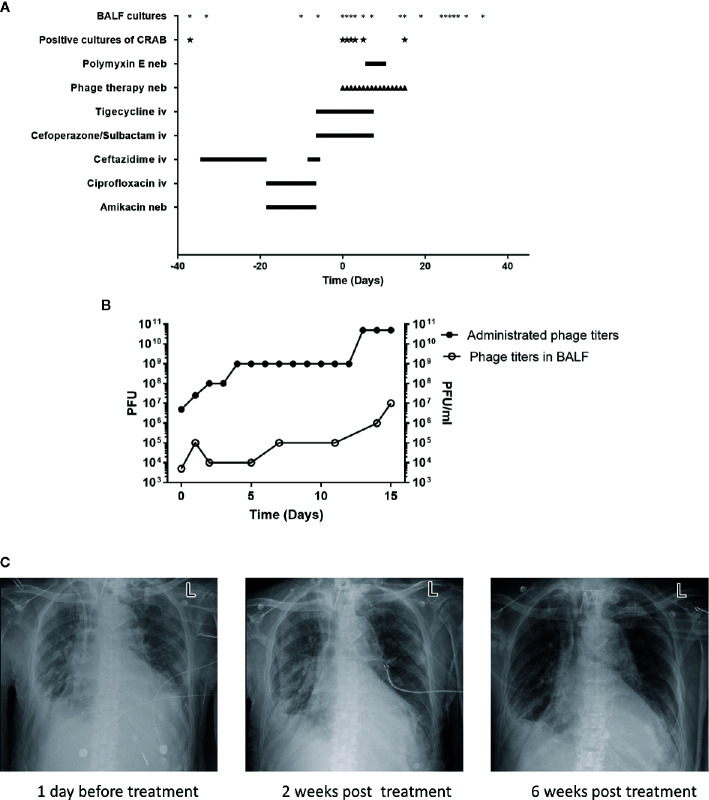
Patient clinical data in phage therapy. **(A)** Timeline of bronchoalveolar lavage fluid (BALF) cultures, drug, and phage administration. BALF samples were collected as indicated (*), positive cultures of carbapenem-resistant *A*. *baumannii* (CRAB) have been marked (★). The timeline shows the administration of antibiotics and phage preparation, and the administration of drugs is indicated (day 0 indicates the time when phage therapy was initiated). Iv, intravenous; neb, nebulization. **(B)** Administrated phage titers and phage titers in BALF detected by plaque assays following the phage therapy. Left Y-axis represents the administrated phage titers and right Y-axis represents the phage titers in BALF. **(C)** Chest X-rays results on -1st day, 14th and 42nd day of phage treatment, respectively.

In May 2020, the patient showed signs of lung infection. After almost one month of different episodes of antibiotic therapy (with ceftazidime and ciprofloxacin/amikacin) (**Figure 1A**), the patient’s condition was aggravated. Importantly, in June 2020, the CRAB-positive result of the bronchoalveolar lavage fluid (BALF) culture of this patient drew our attention (**Figure 1A**). The CRAB strains were resistant to all tested antibiotics, except tigecycline and polymyxin E (**Table S1**). Based on these facts, it seemed that the lung infection was most likely caused by this CRAB strain. However, the concentration of tigecycline in the lung is often relatively low (Ramirez et al., 2013). Besides, the patient showed reduced renal function (creatinine clearance at 32.38 ml/min, a normal level is 85–125 ml/min for a healthy male) and the rates of nephrotoxicity with polymyxin E are high (Zavascki and Nation, 2017). These facts indicated that these two antibiotics were not the best choice for the treatment any longer.

Phage therapy is a promising approach for infections caused by multidrug-resistant (MDR) pathogens, several examples of the successful treatment of infections caused by CRAB have been reported (Schooley et al., 2017; LaVergne et al., 2018). Hence, phage therapy was initiated upon the local hospital ethics committee’s approval and the family’s consent for this experimental treatment.

### Phage Isolation and Characterization

**Figure 2:**
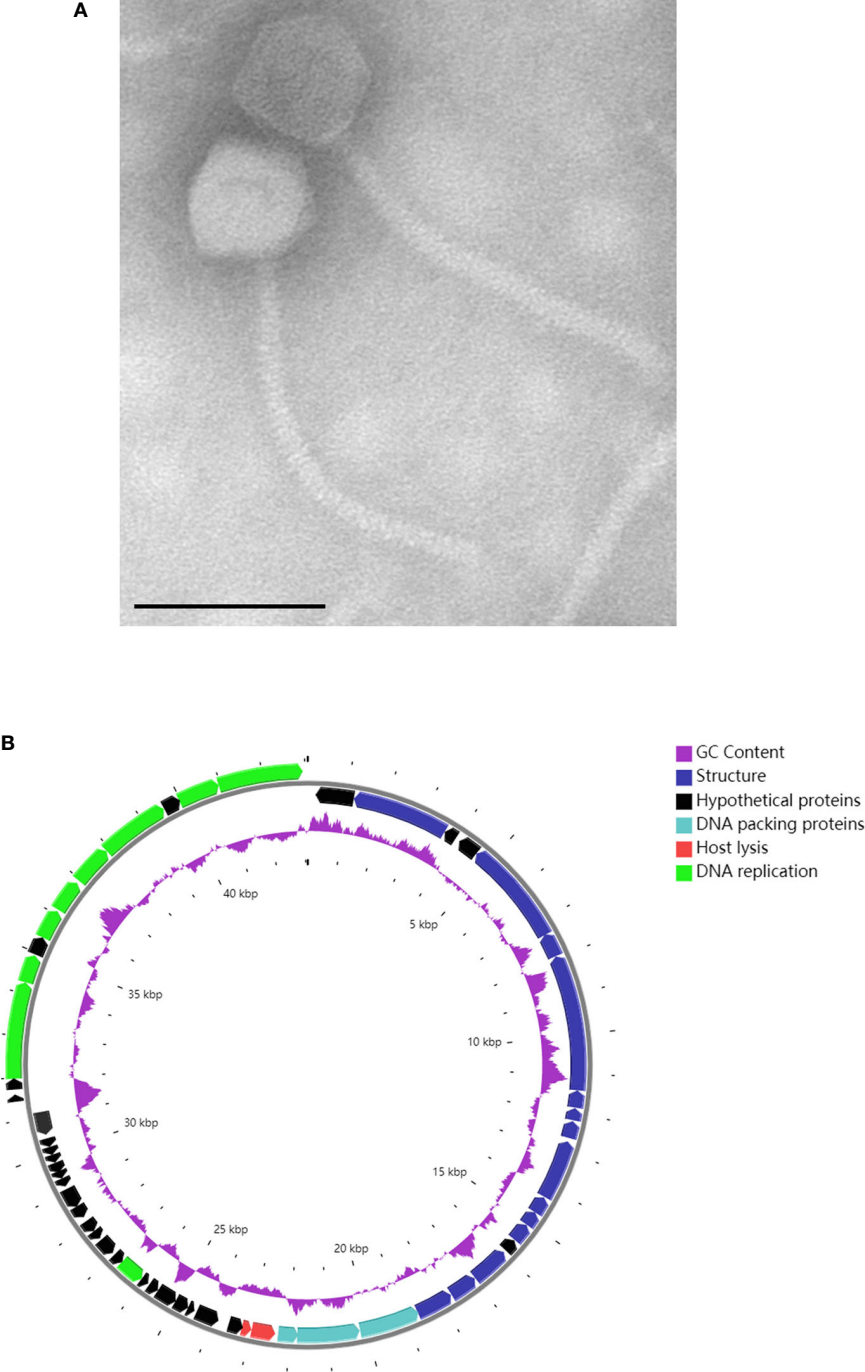
Characterization of phage Ab_SZ3. **(A)** Transmission electron micrographs of the phage. Scale bar represents 100 nm. **(B)** Genome map of the phage. The color of the ORFs refers to five modules: phage structure, blue; host lysis, red; DNA packaging, light blue; DNA replication, green; and hypothetical proteins, black. GC content, purple.

In mid-June 2020, the *A. baumannii* isolates obtained from the BALF of the patient were sent to the lab of Shenzhen Institutes of Advanced Technology, Chinese Academy of Science for preparing a personalized, lytic phage preparation against the MDR *A. baumannii* strain. Ten *A. baumannii* phages *de novo* isolated from different sewage samples showed antibacterial activity. The phage candidate, Ab_SZ3, which showed the strongest antibacterial activity (according to the clearing of the lysis zone) as measured by spot assay was selected for inclusion in the therapeutic phage preparation (see **Figure S1**). Transmission Electron Microscopy studies were performed on the phage preparation and revealed a homogeneous population of phage particles belonging to the *Siphoviridae* morphological group. The virion revealed an icosahedral head structure of 62.2 nm and a contractile tail of 193.2 nm x 8.4 nm (**Figure 2A**).

### Genome Sequence Analysis of Phage Ab_SZ3

The sequence of phage Ab_SZ3 has been deposited in the Genbank databases under accession number MW151244. The length of the phage’s genome was 43,070-bp. Sequence-analysis indicated that it is affiliated to the *Caudovirales* order, *Siphoviridae* family, *Lokivirus* genus. This phage shared the highest identity (99% at the nucleotide level) with *Acinetobacter* phage vB_AbaS_D0 (GenBank: MK411820) and IME_AB3 (GenBank: KF811200).

The circular genetic map of Ab_SZ3 can be seen in **Figure 2B**. Analysis of the phage’s whole genome shows that it possesses a series of genes encoding common phage-related features, including DNA polymerase, DNA helicase, tail, and head structure proteins. It also possesses two genes encoding the host lysis protein, endolysin, and holin. Importantly, the *in-silico* analysis did not reveal any putative virulence, antibiotic resistance, or integrase sequences in the genome of the phage.

### Phage Therapy

Due to the sudden deterioration of the patient’s condition, 50 mg tigecycline was given intravenously twice daily for 6 days before the phage therapy. From 16 July 2020, the personalized phage preparation was nebulized using a vibrating mesh nebulizer (Aerogen) to the patient every 12 h, except for the first 2 doses, which were given once daily. Administrated phage dose was gradually increased: day 0 at 5x10^6^ plaque-forming units (PFU), day 1 at 2.5x10^7^ PFU, day 2 at 10^8^ PFU, day 4 at 10^9^ PFU, and day 13 at 5x10^10^ PFU (**Figure 1B**). Intravenously tigecycline was given twice daily during the first 5 days of the phage therapy, then 50 million IU polymyxin E was administered by inhalation (separately from phage) twice daily for the subsequent 5 days. Antibiotic therapy was completely abolished in the following 6 days. The phage therapy lasted for 16 days in total (**Figure 1A**). Phage and bacterial titers in the lung were monitored by collecting BALF samples, and the inflammation factor profile was monitored by collecting blood samples. Phage particles were detected in BALF 1 h after the treatment started and reached with a titer of 5x10^3^ PFU/ml; phage titer gradually increased in BALF and had a relatively high phage titer (10^7^ PFU/ml) in 15 days. This observation was consistent with the fact of increased administration dose of the phage (**Figure 1B**). Besides, it also suggested phage replication *in situ*.

Phage-resistant bacteria were isolated from BALF samples on day 2 and day 3 after phage therapy. However, it is plausible that phage-resistance could have been associated with fitness trade-offs.

Remarkably, on the 7th day of the phage therapy, for the first time, a culture of BALF did not yield any CRAB. From then on, and until the time of writing (January 2021), except for one culture of BALF on the 15th day of the phage therapy that was positive for CRAB (the isolate remains susceptible to phage Ab_SZ3, see Supplementary Material), all cultures from the patient’s sputum/BALF were negative for CRAB (**Figure 1A**).

**Figure 3:**
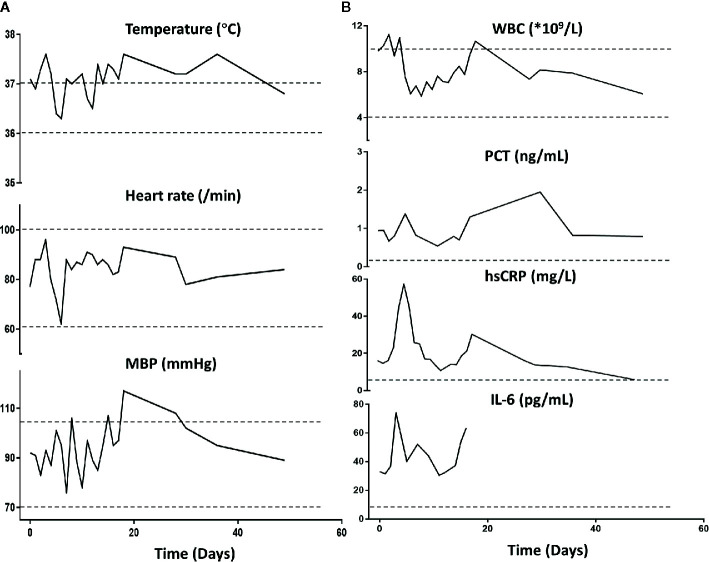
Patient’s clinical examination **(A)** and inflammatory factor profile **(B)**. No major alterations were observed (day 0 indicates the time when phage therapy was initiated). MBP, Mean blood pressure; WBC, white blood cells; PCT, procalcitonin; hs-CRP, high-sensitivity C-reactive protein. Normal ranges are presented between dotted lines in temperature, heart rate, MBP, and WBC; normal ranges are below the dotted line for PCT, hsCRP, and Il-6.

Importantly, the patient restored sinus rhythm on the 8th day after the phage therapy. Furthermore, the bilateral consolidations shown on pre-treatment chest X-rays (**Figure 1C**) gradually disappeared and the lung function improved. Additionally, the inflammation factor profile and the clinical signs of the patient showed no significant side-effect after phage therapy (**Figure 3**).

### Patient Follow-up

In 1 month following phage therapy interruption, the patient was still hospitalized due to the difficulty in weaning from mechanical ventilation. The patient developed 1 episode of sepsis in August 2020, blood culture was positive for *Enterococcus faecium* and *Staphylococcus haemolyticus*, vancomycin was subsequently administered for 15 days. The patient also had 1 episode of a non-MDR *Pseudomonas aeruginosa* strain colonization during this period, although multiple BALF cultures were positive for *P. aeruginosa*, no clinical signs of respiratory infection were observed. Due to the patient’s history of recurrent respiratory infections, amikacin was applied for the decolonization of the *P. aeruginosa* strain (see **Figure S2**). Despite the prolonged, antibiotic-driven selective pressure on the patient’s lung microbiota and the protracted hospitalizations, we did not observe a reappearance of the CRAB. Till the time of writing (January 2021), the patient remains in stable condition.

## Discussion

To ensure the patient’s safety, we started the phage therapy with relatively low phage concentrations compared to other studies (LaVergne et al., 2018; Dedrick et al., 2019). Due to the urgent need for efficacious products for the patient, we undertook a fast way to prepare the personalized phage preparation, in which the endotoxins level remained relatively high (10^5^ EU/ml). However, we took the purified phage preparation (endotoxin levels at 1.3 x 10^4^ EU/ml) for treatment in the days that followed. Under the FDA-recommended endotoxin lipopolysaccharide limit of 5 EU/kg body weight per hour (LaVergne et al., 2018), we gave a relatively low dose of phages initially. Despite this, the endotoxin level in the phage preparation did not stricly follow the FDA recommendation. Our preparation was given by respiratory route instead of the intravenous route. Interestingly, the systemic inflammation factor profile and the clinical signs of the patient showed no significant side-effect after phage therapy. Our result indicates that a higher endotoxin level of the phage preparation for the respiratory route was tolerated in this COPD patient. However, these observations were not consistent with another study, where inhalation of nearly 5 x 10^4^ EU of purified endotoxin were initially well tolerated by COPD patients, who then presented slightly increased systemic inflammation profiles at 24 h (Gupta et al., 2015). We presume that a non-significant change of systemic inflammation after exposure to the relatively high level of endotoxin could be due to this patient’s special condition: age at 88-year-old and history of recurrent infection. Nonetheless, the standards of endotoxin level in phage preparation for clinical use require further investigation.

One of the major concerns regarding phage therapy is that phage-resistant bacteria might cause the failure of phage therapy (Oechslin, 2018). Herein, it is plausible that phage-resistance is associated with severe growth defect. Indeed, the trade-off between phage-resistance and fitness, such as antibiotic susceptibility or growth rate, has been reported elsewhere (Le et al., 2014; Gordillo Altamirano et al., 2021). In our opinion, the emergence of phage-resistance is an argument supporting the effective action of phages at the site of infection, since it implies that phages were able to provide a sufficiently-strong evolutionary pressure. This patient also received antibiotics that the strain was sensitive against during the phage therapy, we cannot exclude the role of antibiotics in the eradication of this pathogen. However, patients with similar clinical conditions typically have high morbidity and mortality under antibiotic monotherapy (Inchai et al., 2015). We also observed evidence suggesting phage replication *in situ*, as well as emergence of phage-resistant mutants. We therefore believe that phage therapy contributed to the clinical improvement of the patient.

In summary, this case demonstrates the clinical efficacy and safety of phage therapy in combination with antibiotics in the treatment of CRAB lung infection with COPD. However, more questions in molecular mechanisms (such as phage receptor, phage resistance, and immunogenicity of phage) involved in phage therapy remain unclear, and phage therapy deserves further evaluation in well‐designed clinical trials in the era of increasing antimicrobial resistance.

## Data Availability Statement

The datasets presented in this study can be found in online repositories. The names of the repository/repositories and accession number(s) can be found below: https://www.ncbi.nlm.nih.gov/genbank/, MW151244.

## Ethics Statement

The studies involving human participants were reviewed and approved by The Shenzhen People’s Hospital Medical Ethics Committee. The patients/participants provided their written informed consent to participate in this study.

## Author Contributions

XT, HC, WH, and YM conceived and designed the study. XT, HC, and MZ performed the experiments and analysis. MZ and WH contributed with data and analysis. XT wrote the manuscript, with contributions and comments from all authors. All authors contributed to the article and approved the submitted version.

## Funding

This work was supported by the Ministry of Science and Technology of China (2018YFA0903600), the Guangdong Provincial Key Laboratory of Synthetic Genomics (2019B030301006), the Shenzhen Key Laboratory of Synthetic Genomics (ZDSYS201802061806209), the Shenzhen Peacock Team Project (KQTD2016112915000294), the International Collaborative Research Fund (GJHZ20180413181716797), the Free Inquiry Fund (JCYJ20180305163929948), the Shenzhen Key Medical Discipline construction Fund (SZXK045), and the Health Commission of Guangdong Province (A2019501).

## Conflict of Interest

The authors declare that the research was conducted in the absence of any commercial or financial relationships that could be construed as a potential conflict of interest.
