# Supplementary material for: Clinical Experience of Personalized Phage Therapy Against Carbapenem-Resistant Acinetobacter baumannii Lung Infection in a Patient With Chronic Obstructive Pulmonary Disease
Source: Front Cell Infect Microbiol. 2021 Feb 26;11:631585. doi: 10.3389/fcimb.2021.631585 (PMC7952606; doi:10.3389/fcimb.2021.631585)
Supplement: Supplementary file 1 [file DataSheet_1.docx]

**Supplementary Materials**

A


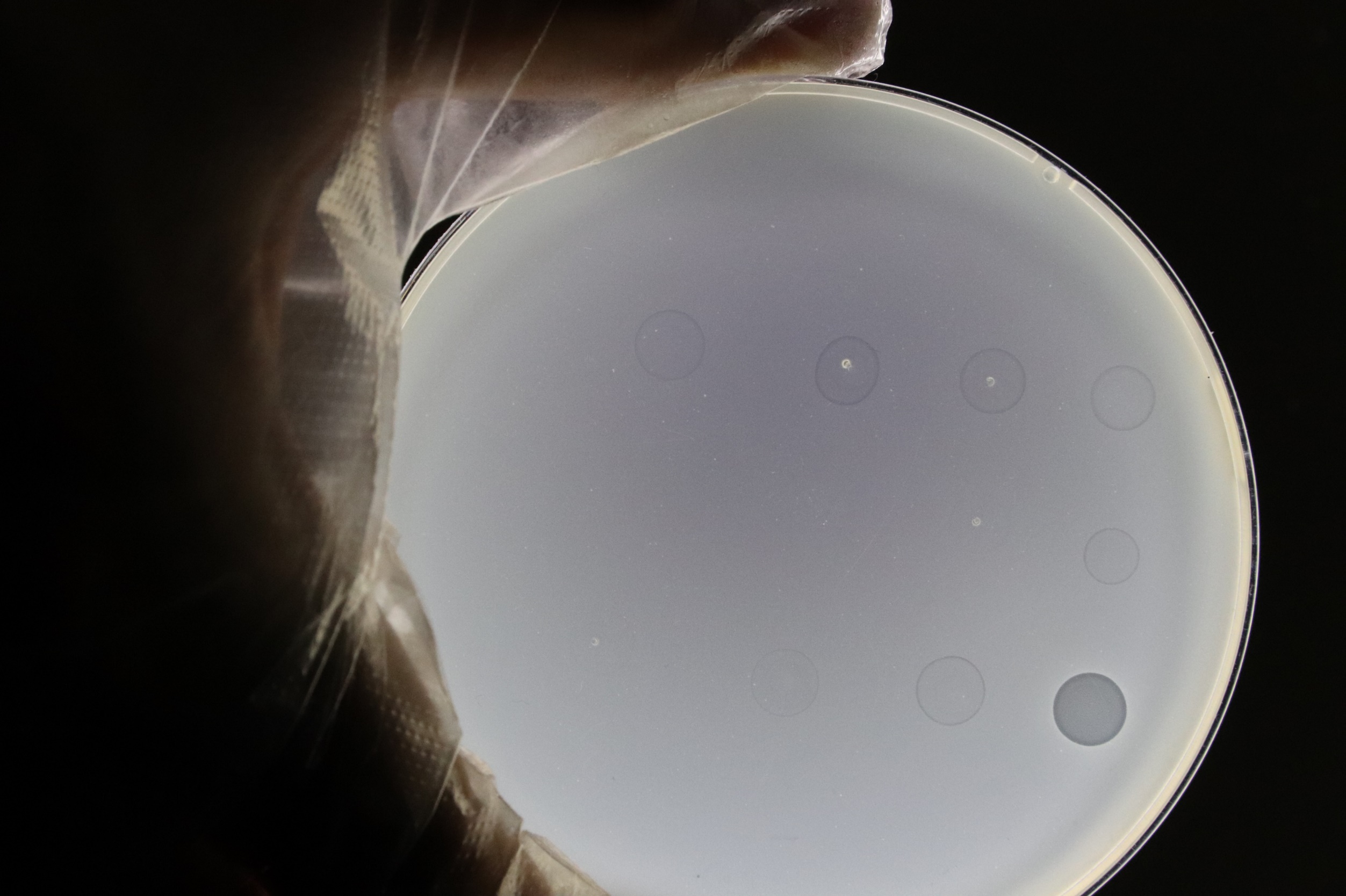


Ab_SZ3

Ab_SZ4

Ab_SZ1

Ab_SZ6

Ab_SZ2


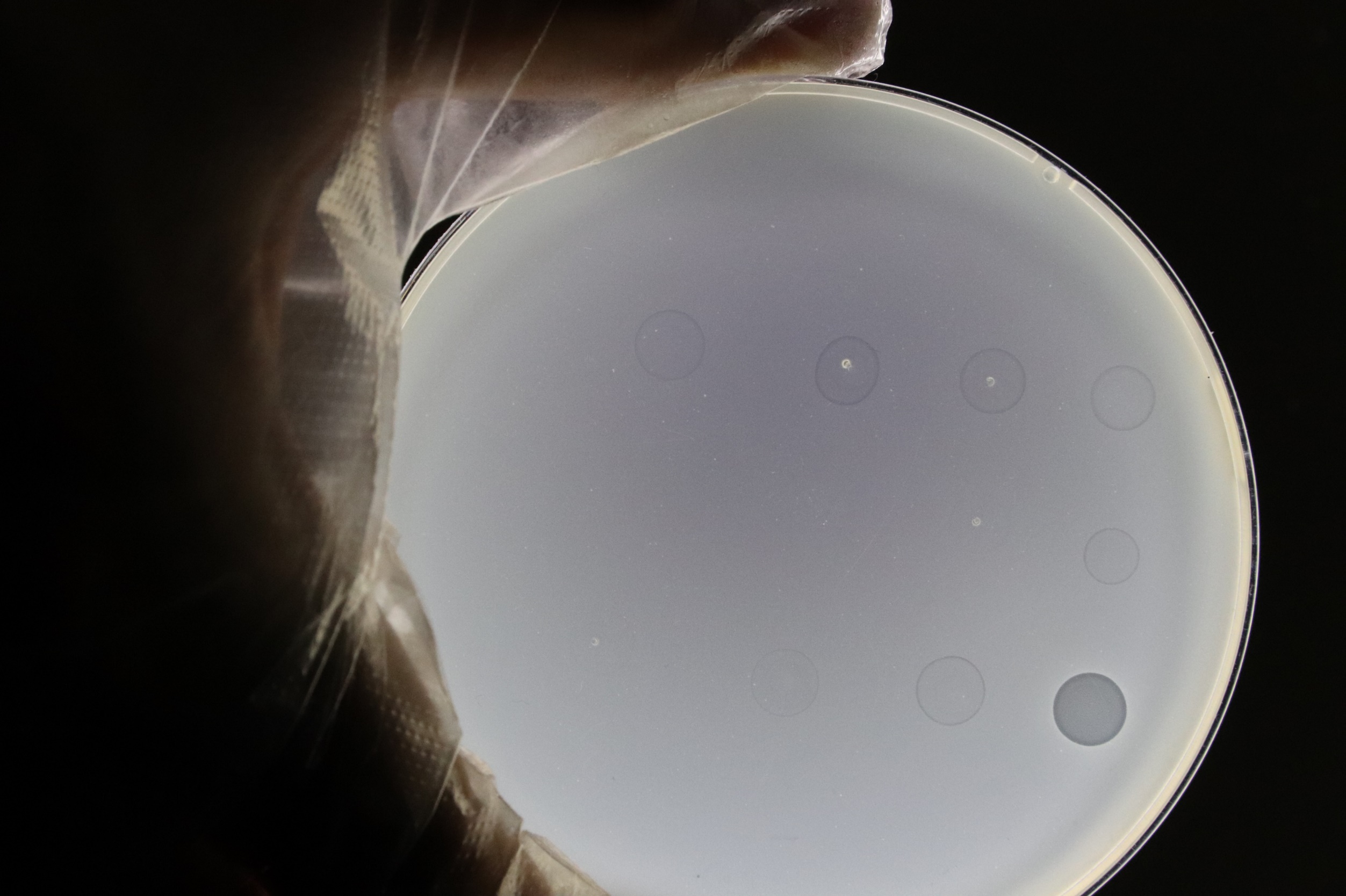


Ab_SZ5

**
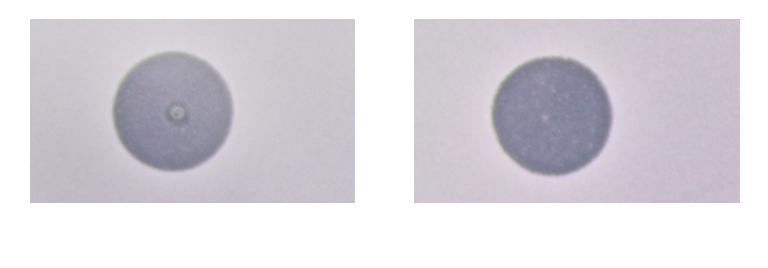
**

B

day 15

day -37

Figure S1. Spot assay of isolated phages on *A. baumannii* clinical isolates. A. Isolated phages (representative phages were chosen, included Ab_SZ1, Ab_SZ2, Ab_SZ3, Ab_SZ4, Ab_SZ5, and Ab_SZ6) were spotted onto bacterial lawns, Ab_SZ3 phage showed the most clearing lysis zone; B. Ab_SZ3 phage was spotted onto bacterial lawns: recovered *A. Baumannii* clinical isolate at day -37 and day 15). These assays were repeated three times with similar results; a representative experiment is shown (day 0 indicates the time when phage therapy was initiated).

Figure S2. Timeline of clinical samples cultures and drug administration after phage therapy. Positive cultures of PA were indicated (*), positive blood culture had been marked (★). PA: *Pseudomonas aeruginosa*.


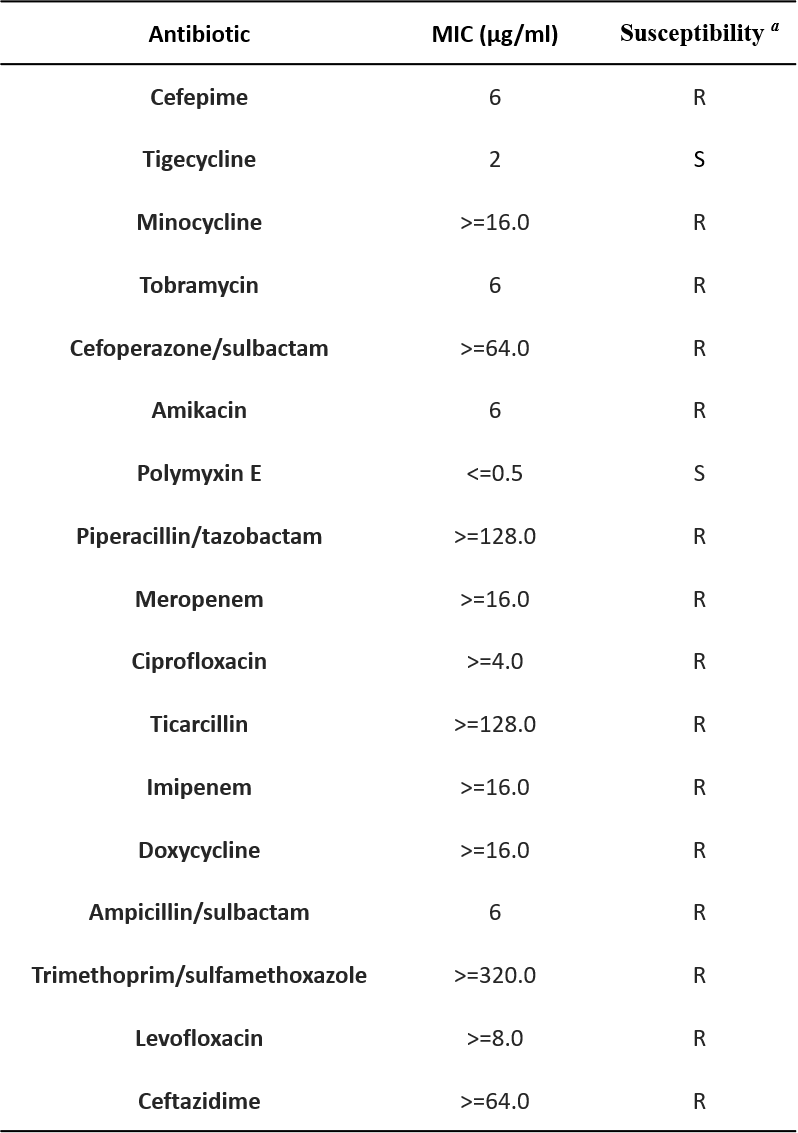


Table S1. Antibiogram of the carbapenem-resistant *Acinetobacter baumannii* strain*.* ***^a^*** S, susceptible; R, resistant (according to CLSI methods).

***^a^*** S, susceptible; R, resistant (according to CLSI methods)
